# Abstracts and Gleanings

**Published:** 1877-07-20

**Authors:** 


### Abstracts and Gleanings.

INDIGESTION.—Dr. Lewis Smith, in the *Virginia Medical Monthly*, says: The following treatment has, in my practice, probably relieved nine-tenths of those cases of dyspepsia, which were not due to organic diseases:

R. Bismuthi subcarbonatis.....dr.ij.

Pepsini (vel Lactopeptini)..dr.iss. Misce.

Divide in crustulas, No. xij. Signe: Take one wafer before each meal, and twenty drops of the following in wine or water after each meal:

R. Tincturæ nucis vomicæ,
Acid muriatic; (adult)....aa.oz.ij. Misce.

In cases attended by constipation and eructation of gas, the following will be found useful:

R. Pulveris carbon. ligni.
Magnes. calcinat......aa oz.j.
Pulveris rhei.... .....oz.ij.ad oz ss.Misce

S. Take half a teaspoonful to one teaspoonful in simple syrup or any convenient vehicle, three times daily. Of course, whatever the medicines employed proper directions should be given in regard to the diet of dyspeptics.

CONSTIPATION.—In habitual constipation of the adult, in which the use of fruits and the most laxative articles of food often has little effect in producing evacuations, the following will be found very efficient, while its purgative effect is not severe, and is commonly without pain:

R. Ext. belladonnæ........gr.iij.
Ext. nucis vomicæ .....gr.vj.
Podophyllin............gr.vj—ix.
Ext. aloes........... ..gr.xviij. Misce.

Divide in pilulas No. xviij. S. Take one when required.

The habitual constipation of infants is a common and troublesome complaint. It can sometimes be remedied when a wet nurse is employed, by the change from one nurse to another, and often by giving a little oatmeal one or more times daily. It is better to employ enemata of water, or water with sweet oil and molasses for habitual use, than to employ even the mildest preparations of those purgative drugs which are in ordinary use, and which produce catharsis by their stimulating or irritating effect upon the surface of the intestines, since the irritation which they cause is apt to impair the function of the gastro-intestinal mucous membrane; or the intestines may become so accustomed to them, that it will be found necessary to increase the dose in order to obtain the desired result.

INFANTILE DIARRHŒA.—The treatment of this disease by small doses of calomel, combined with Dover's powder, has been very generally and properly discarded in New York. The more intelligent physicians prescribe opium and bismuth, with or without pepsin or lactopeptine, and sometimes in combination with chalk. The following prescriptions have been largely and successfully employed in the New York Infant Asylum, and in private practice:

R. Tinct. opii...............gtt. xvj.

Bismuth subnitrat........dr. ij.

Syr. simplic............ .oz ss.

Mistur. cretæ.............oz. iss. Misce.

Give one teaspoonful every three hours to a child of one year.

R. Tinct. opii...............gtt. xvj.
Bismuth. subnitrat........dr. ij.
Pepsini (vel Lactopeptini)..oz. iss.
Syr. zingiberis,
Aq menth peperit....... .aa oz. i.

To be administered in the same dose as the foregoing. In severe cases the dose may be given for a time every two and a half hours.

VOMITING IN CHOLERA INFANTUM. Vomiting is often a prominent symptom in this malady. It sometimes commences before the diarrhœa, and often continues after the latter ceases. It may be controlled by the above prescriptions, and often, also, by lime water given in an equal quantity of milk, to which, double or treble as many drops of Bourbon whisky or brandy are added as the infant is months old. A few drops of chloroform, in cold water, will also sometimes control the vomiting. Carbolic acid, given in doses of 1-10th to ⅙th of a drop has been recommended by writers for the nausea, but I have not observed any decided benefit from its use in the majority of instances in which I have had an opportunity to witness its effects. But there is another remedy which I can recommend, which is seldom used for this purpose, and the dose of which is so small, that most physicians will probably think it inert, namely: 1-10th to ⅓th of a drop of tincture of ipecacuanha, given to the infant in a teaspoonful of cold water, every hour or second hour, till the nausea ceases. The reports of its use in two of the institutions of New York have been favorable. A physician of New York, exact in his observations, and cautious in his statements, has informed me that he recently relieved vomiting in an adult, when other remedies had failed, by one drop doses of the same medicine.

Black Haw in Abortion and Dysmenorrhœa.—Professor E. W. Jenks remarks of this agent:

Where the habit of aborting has been formed, my mode of prescribing the viburnum is, to have the patient take from half a teaspoonful to a teaspoonful of the fluid extract four times a day, beginning at least two days before the regular menstrual date, and continuing it not only during the usual period of the catamenial flow, but two days longer than that discharge continues when the woman is not pregnant. Where there are indications that an abortion is imminent, the fluid extract can be administered in teaspoonful doses every two or three hours as long as its use seems to be demanded.

The writer would designate viburnum prunifolium as a uterine sedative, whose action is as pronounced, as is that of ergot in causing uterine contraction. It is not alone in the prevention of abortion that it proves, by virture of this peculiar sedative action, a most valuable therapeutic agent. It proves equally efficient in the treatment of the sympathetic disorders incident to pregnancy, where a nervine or sedative is demanded, and in a large class of non-puerperal diseases of women. The use of viburnum in this last mentioned class of cases deserves more attention than it has heretofore received, and will occupy the remaining portion of this brief paper.

It could not be otherwise, than that a remedy which is known to exercise such a potent effect upon the pregnant uterus, must be of much service in many affections of the non-pregnant woman. I am convinced, from an experience in its use extending now over more than six years, both in private and hospital practice, that viburnum is a valuable acquisition to the gynecologist's list of remedies.

I would give as a general statement concerning the uses of viburnum, that it is serviceable in all uterine disorders characterized by loss of blood.

In menorrhagia, or metrorrhagia, depending wholly upon systematic causes, as *e. g.* that in phthisis, organic diseases of the heart, hepatic disorders, anæmia, or malarial diseases, it is peculiarly applicable. There is no depressing effect succeeding its administration; on the contrary it is a grateful tonic, serving to stimulate rather than depress. Patients for whom I have prescribed it without informing them for what purpose, have repeatedly spoken of its pleasant, stimulating effects. In the metrorrhagia incident to the menopause with the multiplicity of nervous derangements from which women suffer at this period, viburnum has proven very beneficial. It will modify the hæmorrhage from such causes, where ergot in full doses is not well tolerated, or where the patient is in a feeble condition, I have been in the habit of combining the two remedies in various proportions, with gratifying results. I have never known painful uterine contraction to be produced by viburnum alone, nor do I think that oxytoxic effects can be attributed to it.

Viburnum is serviceable also in certain forms of dysmenorrhœa. My attention was first directed to its worth in this affection several years ago, by the remark of a patient for whom I had prescribed it for a profuse menstrual discharge. She said that she had taken the medicine during the menstrual periods, beginning two or three days before each, and that there was not only a diminution in the quantity of blood, but that menstruation was more nearly painless than it had been before for years. This remark was suggestive, and I have since given viburnum alone such a thorough trial in the various forms of dysmenorrhœa, that I have arrived at the following conclusions: In all forms of dysmenorrhœa attended with profuse menstruation, viburnum, if administered for several days in advance of the period, as well as during the continuance of the discharge, affords the patient great relief. Where there is with the dysmonorrhœa a scanty flow, it does not prove beneficial. If the pain is produced by stenosis, or any mechanical obstruction, it affords only moderate relief. It is not sufficiently sedative, if given alone, to fully relieve the sufferings of spasmodic or neuralgic dysmenorrhœa. It is, however, a valuable adjuvant to the sedative and anti-spasmodic remedies, such as cannabis indica, camphor, hyoscyamus, and conium.

In that form of dysmenorrhœa with menorrhagia, caused by fibroid growths impinging upon and twisting the uterine canal, viburnum, in combination with ergot, has proven beneficial, and much more so than either remedy if given without the other.

Gentiana Quinqueflora.—C. A. Yelvington, M. D., Susquehanna, Pa., says of this plant: My first trials with it were three cases of obstinate intermittent, in which quinine and other anti-periodics had failed to accomplish the desired result; and, to my surprise, in one week's time the use of a decoction of the herb had completely controlled the disease. This led me to try it in similar cases, and with the happiest results, and I am now satisfied that we possess no agent superior to it as an anti-periodic. In antonic conditions of the digestive apparatus, in combination with Hydrastis, I have found it a splendid tonic, increasing the biliary secretion, and acting as a stimulant to the excretory organs. In fevers I have used it in combination with gelseminum with good results; and in derangements of the biliary organs, in combination with leptandrin or podophyllin, it seems to increase the effect of these agents. I have never used it in combination with Veratrum, believing them to be incompatible.

To sum up its qualities, I would say that where an atonic condition of the digestive organs is present I never have found its equal, Also, in derangements of the biliary organs, in combination with the above remedies, I have accomplished great results. Nor is this all my own experience; I have at times furnished it to other physicians, who have informed me that in the same conditions it has proved beyond a doubt a specific where an anti-periodic is indicated. I have given it the place formerly occupied by quinia, and have accomplished better results in less time than I could with that remedy. Having used it for ten years in my practice, and having always accomplished great good with it, I feel that I can recommend it to the profession as a safe and reliable remedy, and one that will prove a great aid in their practice.—*Eclectic Medical Journal.*

Balsam of Tolu as an Application to Wounds.—At a meeting held October 11, 1875, of the Berliner medicinische Gesellschaft, Dr. E. Wiss spoke in almost unbounded praise of balsam of tolu as an application to wounds of all kinds. When the balsam was put upon wounds it produced an immediate sensation of burning, which, however, very soon ceased, as did all pain, even in most severe wounds. Fresh wounds under this treatment showed no inflammation, and in those already inflamed it soon ceased. No suppuration took place, and where it was already present it soon disappeared. No wound treated by him by this method took on a septic character, even under the most unfavorable local and climacteric surroundings. In all cases, even in lacerated wounds, there was union by first intention, a thing which had not been his experience in any other method of treatment. Two cases were detailed. He considers that the balsam hinders suppuration, and after his surgical experience he made use of it in two cases of old women with profuse catarrh of the lungs. The drug was given in an emulsion with the yolk of egg (4.0:120.0=64.8 drops to 4 oz.; one teaspoonful every two hours), and one case was well in eleven days, while the other recovered in three weeks. In these cases "all other medicines had failed."—*Boston Medical and Surgical Journal.*

Bilious Attacks.—Dr. Fothergill (in *Medical Times*) says of the treatment of bilious attacks to which dark-complexioned persons of the biliary diathesis are most subject: Rarely do persons of other diathesis and fair persons suffer from those disturbances which may fairly be said to be connected with the presence of bile acids in excess; while as to those forms of biliary disturbance where the urine is laden with lithates, the condition Dr. Murchison calls lithæmia, persons of other diathesis seem equally liable to them, and they are found in fair and dark people alike. For those bilious attacks, then, which occur chiefly in those of the bilious diathesis, nothing is so good as alkaline saline purgatives taken in some vegetable infusion immediately on getting out of bed in the morning. This should be washed down with some warm fluid which excites the peristaltic action of the bowels, and, if necessary, a vegetable laxative pill should be taken the night before. After a couple of liquid motions—the more copious the better—the bilious person feels pretty equal to the day's work before him. Rochelle salts with a little sulphate of magnesium in infusion of buchu forms a most excellent morning purge, in my experience. Sir Joseph Fayrer has found in his Indian experience sulphate of magnesium, with quinine or gentian, sufficient to produce two or three loose motions, an efficient measure in biliary congestion.

Use of the Obstetrical Forceps.—Prof. S. D. Turney, in a paper read before the Central Ohio Medical Association, on the use of forceps, concludes with the following propositions:

1. That the forceps are, in competent hands, absolutely safe for mother and child.
2. There being neither danger to mother nor child, they may be applied at any time in the second stage of labor; when the fœtal head ceases to advance, or it is necessary to hasten the delivery.
3. That the long are preferable to the short forceps.
4. That in their application reference should be had more to the pelvis of the mother than to the head of the child.
5. That when the head of the child is at the superior strait, or in the cavity of the pelvis, traction *may be* made during the pain, should pain exist; but when the head is pressing upon the perinæum the rigidity and elasticity of this organ are more safely overcome by a force continuously applied, and, therefore traction should be made in the interval and not during the continuance of the pains.—*Ohio Med. and Surg. Journal.*

Anæsthesia Produced by a Hypodermic Injeciton of Bromohydrate of Quinine.—M. Thaon, of Nice (*Le Mouvement Med.*, 1877, page 153), had under his care a case of intermittent fever which resisted the administration of bromhydrate of quinine administered by the stomach, and also hypodermically, ten to fifteen grains of the salt being given daily, for some days without having mastered the fever. One of the punctures of the hypodermic needle, however, which had been made into the forearm in the region of one of the ante-brachial branches of the musculo-cutaneous nerve, caused complete anæsthesia of the skin over a belt twelve centimetres (four inches) long by six centimetres broad. Thermo-anæsthesia extended a little farther. One month subsequently this anæsthesia remained persistent.—*Medical Times.*

Treatment of Spasm of the Glottis. The attacks of spasm of the glottis are much more violent than those of false croup, being accompanied by contraction of the muscles of respiration, especially of the diaphragm, and sometimes even by general convulsions. In the treatment of this affection there is rarely time to employ the various methods mentioned in the books, such as electricity, frictions, chloroform, etc., and consequently the plan proposed by M. Charon seems to be all the more practicable. This physician states that inhalations of ammonia rarely fail to cut short the attack. He advises mothers who have children subject to attacks of spasm of the glottis, always to carry a bottle of ammonia with them. He cites the case of the wife of a physician, who followed this advice, and whose child always rapidly recovered from the spasm with the help of the ammonia. Unfortunately, one day she did not have her flask with her, and while she was looking for it, the child died asphyxiated.—*The Medical Record.*

Celluloid.—This substance, though prepared by Mr. Hyatt, an American, as long ago as 1869, has only lately been turned to much practical account. It is prepared by subjecting ordinary paper to the action of a mixture of nitric and sulphuric acids; washing this till all trace of acid disappears; drying the product, powdering the same, and mixing it with camphor; drying and repeatedly pressing this mixture, at last applying heat, when the celluloid appears in the form of transparent, elastic rods or slabs. As it is hard, and not easily broken at ordinary temperature, susceptible of high polish, and capable of being cut into extremely thin plates, yet elastic, and, at high temperatures, malleable, plastic, and even fusible, it has become extensively used in the manufacture of the rims of eye-glasses, cheap ornaments, cigar cases, etc., and, when colored, as a means of imitating ebony, lapis lazuli and malachite. It has also been employed in making elastic belts, trusses, etc., and some of its applications in dentistry were patented as early as the year of its discovery.—*Monituer des Produits Chim.*

Who Discovered Anæsthesia?—The claimants—Wells, Jackson and Morton—are well know, and volumes have been written for and against their claims. Less has been known of the fourth claimant, Dr. Crawford W. Long, of Athens, Ga. From testimony recently produced, it does appear that Dr. Long has better claims to be honored as the discoverer of anæsthesia than either of the others. The proofs that he did produce the condition as early as March 20, 1842, appear to be satisfactory and conclusive. Dr. J. Marion Sims, who has recently written a pamphlet setting forth the facts regarding Dr. Long's experiments, presents his claims with an array of evidence which is irresistible. Dr. Long is still living, and in poverty, in Georgia, having lost his all in the civil war. He is represented as being worked to death to obtain his daily bread; he is old and feeble. The fate of Wells, Morton and Jackson is sad indeed. Poor Morton, worn out with disappointment and grief, became insane, and so injured himself in an asylum as to cause his death. Wells became insane, and committed suicide in New York in 1848. Jackson, so long known as Boston's distinguished chemist, is now in an insane asylum, hopelessly incurable. What a sad, pitiable record is this!

Dr. Long alone remains (of the four who have contended for the honors of the discovery of anæsthesia) in the possession of his reason, and he is destitute, overworked, disheartened. And now, what can we do, what shall we do, in justice to all? Dr. Sims' suggestion is a good one. Let the whole medical profession, North, South, East, West, unite in asking Congress, at its next session, to appropriate the sum of four hundred thousand dollars to be divided equally among the families of Wells, Morton, Jackson, and Long.

The discovery of anæsthesia is the greatest boon yet conferred upon man, and it is an American discovery. Let us all join in one united effort to aid those who have suffered so much in connection with this discovery.—*Boston Journal of Chemistry.*

The Feeding of Infants.—Dr. W. Faussett, in an interesting article on this subject in the *London Medical Press and Circular*, arrives at the following conclusions:

1. That aliment should always be presented to the infant stomach in a perfectly fluid form.

2. That as bread and farinaceous substances generally have been proved by experience, and recently by numerous post-mortem examinations, to be often indigestible, and to have led directly to infant mortality, such substances had better be excluded from infant feeding.

3. That cow's or goat's milk, when pure and modified as much as possible, to resemble human milk, will often be found sufficient, without any other help, to nourish the new-born infant

4. That as cocoa contains all the elements indispensable for the growth and development of the body, and can always be presented in a fluid form, it is, next to milk, preferable to all other natural substances as an article of infant aliment.

There is one other point which, though only indirectly connected with infant feeding, is one of paramount importance, as regards the present and future health of the individual, namely, the necessity of guarding against the hateful practice of covering the child's face as it sleeps. *Boston Journal of Chemistry.*

Albumen in the Treatment of Consumption.—Dr E. L. Shurly (in *Buffalo Medical Journal*) reports remarkable results in the treatment of pulmonary consumption by the use of the albumen of eggs, as the most concentrated and powerful nutriment that can be found. "But finding quite often that patients were unable to digest or assimilate the requisite number of eggs, owing, perhaps, either to the oil contained, or the inability of the stomach to break up the intimate cell structure, I was led to present them in a dessicated form, either entire, or just the whites, with, to my observation, gratifying results. The mode of preparation is as follows: The eggs are exposed to a warm current of air, never above 110° F., until they are thoroughly dried, when the residue will be ready for use. As it is more tedious and difficult to evaporate the whole contents, than the whites, I more often throw out the yolk, and afterwards prepare the albumen by adding a little cod liver or other oil; or triturating it with either egg shell or one of the phosphates, preferably calcium or sodium phosphate. Particular attention must be paid to its preparation, inasmuch as albumen (or dessicated egg) prepared either by too great heat or by precipitation, whereby it is coagulated, will not yield the same results as that prepared in the manner here given, which renders it readily soluble in warm water, and capable of direct absorption as albuminose by the stomach. I have never observed an instance where it was not borne easily by the stomach when prepared by means of gentle heat. It may be administered in teaspoonful doses as a powder with or without one of the salts mentioned, by throwing it in warm water, light soup, or milk, or emulsified with glycerine or cod liver oil."

He gives a number of cases absolutely relieved by this method.

Singular Property of the Tomato Leaf.—"I planted a peach orchard," writes M. Siroy, of the Society of Horticulture, Valparaiso, "and the trees grew well and strongly. They had just commenced to bud when they were invaded by the curculio (*pulgon*), which insects were followed, as frequently happens, by ants. Having cut some tomatoes, the idea occurred to me that, by placing some of the leaves around the trunks and branches of the peach trees, I might preserve them from the rays of the sun, which were very powerful.

"My surprise was great upon the following day to find the trees entirely free from their enemies, not one remaining, except here and there where a curled leaf prevented the tomatto from exercising influence. These leaves I carefully unrolled, placing upon them fresh ones from the tomato vine, with the result of banishing the last insect and enabling the trees to grow with luxuriance. Wishing to carry still further my experiment, I steeped in water some fresh leaves of the tomato, and sprinkled with this infusion other plants, roses, and oranges. In two days these were also free from the innumerable insects which covered them. I felt sure that, had I used the same means with my melon patch, I should have met with the same result. I therefore deem it a duty I owe to the Society of Horticulture to make known this singular and useful property of the tomato leaves, which I discovered by the merest accident."—*Scientific American.*

VARICES OF THE LEG. — Operative treatment of varices of the leg is as old as medicine. New and dangerless is the method of assistance by the antiseptic bandage. Since Dr. Risel, of Halle, has discovered the cause of these ectasia to depend not upon an impermeability of the saphenous vein, but, on the contrary, upon simple hydrostatic pressure, over distension, he has extirpated not only the varicose knots and tortuosities on the leg, but also a piece of the trunk of the vein itself. The region of the varicosities is brought out as fully as possible by means of elastic ligatures, then the veins are dissected free, surrounded freely with catgut, and cut out between the ligatures. Risel cut out in one session, from the same leg, six pieces of vein each 4-5ctm. long. Eleven legs of nine patients were treated in this way, and under strict antisepsis not the least accident occurred in the healing process. The cure was complete, mostly, in eight days so that the patients could walk about. Œdema, thrombosis, etc., occurred in no case. One of the patients, a farm overseer, presented himself after a few months with completely cured legs, cured not only of varicose veins, but also of an old and obstinate eczema. The further histories of the other patients had not been followed up.—*Deutsche Med. Wochenschr.* 1877, No. 8.

IMPACTION OF GALL-STONES.—In the *Canada Lancet*, for June, Dr. Thomas S. Barclay, of Detroit, Michigan, reports a case of "Impaction of Gall-stones and Obstruction of the Bowel," causing death. A post-mortem was made and the gall-bladder was found packed full to distension, with gall-stones, to the number of 700, from the size of a pin's head to a bean. "The systic and common ducts were entirely occluded, and fibrous bands were attached from the gall-bladder to the bowel, causing constriction of the duct. The smallest probe would not enter the common duct, and the bowel would not admit a common quill. The constriction of the bowel extended from the stomach down to the middle of the descending portion of the duodenum. The liver was somewhat enlarged; the heart small and soft, but no valvular trouble. The stomach was perfectly healthy; all the other organs normal." The patient had been subject to attacks of bilious colic, every few months, which would pass off under treatment.

Dr. Barclay remarks: "This case was very interesting, from the fact that there was a difference of opinion among the medical attendants as to the nature of the trouble. This was entirely cleared up by the post-mortem examination. One lesson which may be drawn from the case is, the importance of a careful examination of the fæces for the presence of gall stones, after these so-called attacks of bilious colic. It is very likely that he passed numbers of them from time to time, but finally their accumulation in the gall-bladder, and consequent pressure, produced inflammation, which resulted in what we found after death. I am persuaded that there are more cases of this kind than generally supposed. Within the past three years I have met with no less than twenty-three cases. The succinate of iron has been very successful in my hands in arresting the formation of these stones."—*Maryland Medical Journal.*

ADMINISTERING IODINE THROUGH A NURSE.—Dr. Gemmel, of Birmbaum, relates the case of a feeble, rickety child a year and eight months old, to whom it was thought of great importance that iodine should be administered, which, however, in any form tried, had induced vomiting and irregular action of the bowels. It was then resolved to try giving it through the milk of a nurse, and in a few days after she had begun taking it, her milk was sufficiently impregnated with it. It was found also, that a cow's milk could be similarly affected by giving the animal ten grammes of iodide of potassium per diem for a fortnight. The child under the use of the nurse's milk bore the iodide very well, and soon recovered.—*Berlin. Klin. Woch., April* 9.

DIETETIC PREPARATIONS.—Subscriber (*Chicago, Ill.*) writes as follows: "Will you be kind enough to publish in your valuable journal some simple recipes for preparing the various dietetic preparations, such as are ordered for the sick? I have frequently been asked by customers how to prepare various broths and gruels, and although this query may not be quite in your line I believe many druggists would be pleased to be furnished with reliable recipes.

*Answer.*—We take pleasure in replying to your query, and, as you remark, no doubt many of our readers will be pleased to have a few good recipes for these preparations, a proper knowledge of which is too often neglected by the apothecary, who may frequently be called upon to furnish information as to the manner of preparing them. Indeed they may properly be considered as auxiliaries to medical treatment:

BEEF TEA,

| | |
|---|---|
| Beef, lean, cut in small pieces | 1 pound. |

Put into a jar without any water; cover lightly and set in a pot of cold water. Heat gradually to a boil, and continue this steadily for three or four hours until the juice is all extracted from the meat. Season with salt, and when cold, skim. This may be served hot or cold; but will frequently be preferred by the patient in the latter way.

MUTTON BROTH.

| | |
|---|---|
| Mutton, lean, cut in small pieces | 1 pound. |
| Water, cold | 1 quart. |
| Rice, or barley, soaked in a very little warm water | 1 tablespoonful. |
| Milk | 4 tablespoonfuls. |

Boil the meat in the water until it falls to pieces, keeping the pot closely covered. Strain the liquid off, and add the soaked barley or rice; simmer half an hour, stirring often; season with salt, pepper, and a little chopped parsley, add the milk, and again simmer for a few minutes, taking care that it does not burn.

CHICKEN BROTH.

This may be prepared in the same manner as mutton broth, cracking the bones well before putting in the fowl.

ARROWROOT JELLY.

| | |
|---|---|
| Arrowroot, Bermuda | 2 heaping teaspoonfuls. |
| White Sugar | 2 teaspoonfuls. |
| Lemon Juice | 1 teaspoonful. |
| Water, boiling | 2 quarts. |

Wet the arrowroot in a little cold water, and rub smooth. Then stir it into the pot, which should be over the fire actually boiling at the time, with the sugar already added. Stir until clear, boiling steadily all the while, add the lemon. Pour into a cup, or form, previously wet in cold water.

INDIAN MEAL GRUEL.

| | |
|---|---|
| Indian meal | 1 cup. |
| Flour previously mixed with cold water | 1 tablespoonful. |
| Water, boiling | 2 quarts. |

Wet the meal and flour to a smooth paste, stir into the water while it is actually boiling. Boil slowly half an hour, stirring up well from the bottom. Season with salt to taste. If a laxative is desired, omit the wheat flour.

OATMEAL GRUEL.

This is prepared in the same manner as Indian meal gruel.

TOAST WATER.

Pour sufficient *boiling* water to slightly cover slices of toast, which should be nicely browned. Cover the vessel containing them closely, and let them steep until cold. Strain off the water, sweeten to taste, and add a piece of ice to each glassful.

FLAXSEED LEMONADE.

Flaxseed .......................4 tablespoonfuls.
Lemons, juice of............ ...2.
Water, boiling..................1 quart.

Steep three hours in a covered pitcher. If too thick, add cold water with the lemon juice, and sweeten to taste. Should be iced for drinking.

[We must confess ourselves indebted to Marian Harland's admirable book, "Common Sense in the Household," for the foregoing excellent recipes.]—*Druggists Advertiser.*

NEURALGIA OF FEMALE URETHRA—A desperate neuralgia sometimes afflicts the the female urethra and orifice of the bladder. But very often what seems to be a pure neuralgic affection depends upon minute ulcers in the urethral mucous membrane. By an ingenious contrivance Mr. Ashwell washes the whole tract of membrane with a strong solution of nitrate of silver, and by this plan he cured a very severe case of the disorder. I obtained equal success in an exceedingly obstinate case by the passage of a soft bougie every night and morning.—From John Kent Spender's Therapeutic Means for relief of pain.—*Louisville Medical News.*

FOR ECZEMA.—

R. Elix. iodo bromode calcium com..oz. viij.

Take a teaspoonful in water four times a day.

R. Tinc. benzoin.............. .......oz. i.

Apply on brush night and morning. With this treatment we have succeeded in effecting a cure of one case which had become chronic, and had resisted all medication, covering a period of several years.—*Jour. Mat. Med.*

LACTO-PHOSPHATE OF LIME AS A TOOTH FILLING.—The treatment of exposed dental pulps and sensitive dentine is the subject of an interesting paper by Junius E. Cravens, D.D.S. The lacto-phosphate of lime is applied to the exposed pulp, which is carefully sealed and left undisturbed for several weeks. On removing the coverings a new bone is found, its surface continuous with that of the formerly soft dentine, and the sensibility being even below the normal degree. Oxy-chloride of zinc and other substances, however, may produce like results. *Medical Record.*

